# Predictors of outcomes in geriatric patients with moderate traumatic brain injury after ground level falls

**DOI:** 10.3389/fmed.2023.1290201

**Published:** 2023-12-12

**Authors:** Sebastian Peter Forssten, Rebecka Ahl Hulme, Maximilian Peter Forssten, Marcelo A. F. Ribeiro, Babak Sarani, Shahin Mohseni

**Affiliations:** ^1^Division of Surgery, CLINTEC, Karolinska Institute, Stockholm, Sweden; ^2^Department of Orthopedic Surgery, Örebro University Hospital, Örebro, Sweden; ^3^Division of Trauma and Emergency Surgery, Department of Surgery, Karolinska University Hospital, Stockholm, Sweden; ^4^Department of Orthopedic Surgery, Faculty of Medicine and Health, Örebro University, Örebro, Sweden; ^5^School of Medical Sciences, Örebro University, Örebro, Sweden; ^6^Pontifical Catholic University of São Paulo, São Paulo, Brazil; ^7^Khalifa University and Gulf Medical University, Abu Dhabi, United Arab Emirates; ^8^Department of Surgery, Sheikh Shakhbout Medical City, Mayo Clinic, Abu Dhabi, United Arab Emirates; ^9^Division of Trauma and Acute Care Surgery, George Washington University School of Medicine and Health Sciences, Washington, DC, United States

**Keywords:** ground level fall, traumatic brain injury, geriatric, complications, prediction

## Abstract

**Introduction:**

The elderly population constitutes one of the fastest-growing demographic groups globally. Within this population, mild to moderate traumatic brain injuries (TBI) resulting from ground level falls (GLFs) are prevalent and pose significant challenges. Between 50 and 80% of TBIs in older individuals are due to GLFs. These incidents result in more severe outcomes and extended recovery periods for the elderly, even when controlling for injury severity. Given the increasing incidence of such injuries it becomes essential to identify the key factors that predict complications and in-hospital mortality. Therefore, the aim of this study was to pinpoint the top predictors of complications and in-hospital mortality in geriatric patients who have experienced a moderate TBI following a GLF.

**Methods:**

Data were obtained from the American College of Surgeons’ Trauma Quality Improvement Program database. A moderate TBI was defined as a head AIS ≤ 3 with a Glasgow Coma Scale (GCS) 9–13, and an AIS ≤ 2 in all other body regions. Potential predictors of complications and in-hospital mortality were included in a logistic regression model and ranked using the permutation importance method.

**Results:**

A total of 7,489 patients with a moderate TBI were included in the final analyses. 6.5% suffered a complication and 6.2% died prior to discharge. The top five predictors of complications were the need for neurosurgical intervention, the Revised Cardiac Risk Index, coagulopathy, the spine abbreviated injury severity scale (AIS), and the injury severity score. The top five predictors of mortality were head AIS, age, GCS on admission, the need for neurosurgical intervention, and chronic obstructive pulmonary disease.

**Conclusion:**

When predicting both complications and in-hospital mortality in geriatric patients who have suffered a moderate traumatic brain injury after a ground level fall, the most important factors to consider are the need for neurosurgical intervention, cardiac risk, and measures of injury severity. This may allow for better identification of at-risk patients, and at the same time resulting in a more equitable allocation of resources.

## 1 Introduction

A ground level fall (GLF) is defined as “inadvertently coming to rest on the ground, floor or other lower level” (1). GLFs are responsible for 15% of all emergency department visits in the USA and account for 25–33% of all injuries among the elderly (2–4). The financial burden of fall-related injuries is also substantial, with estimated costs exceeding $30.4 billion in the United States alone (5). The likelihood of experiencing such an event increases significantly with age, particularly among individuals aged 80 years and above (1). Given the growing elderly population worldwide, the incidence of traumatic brain injuries (TBI) from GLFs is expected to rise as well (6, 7), leading to an even greater burden on medical, rehabilitation, and nursing care resources.

While approximately 20% of all hospital admissions for TBI fall under the classification of moderate severity, this particular group of patients has unfortunately been overlooked in research efforts (8). Additionally, limited knowledge exists regarding older adults with TBIs from a population-based perspective, which directly contributes to the absence of clear treatment guidelines for those with moderate TBIs in this vulnerable patient population (9, 10). To enhance care of this patient category, improve quality of life, and decrease costs of care, it is essential to identify factors that can be used to predict complications and mortality as well as mitigate these adverse outcomes, if possible. The aim of this study was therefore to determine the most critical variables for predicting in-hospital complications and mortality in geriatric patients who have suffered a moderate TBI after a GLF. The hypothesis was that these adverse outcomes would in large part be predicted by variables already present at hospital admission. By doing so, the goal is to lay the groundwork for delivering better and more targeted care to this specific patient population.

## 2 Materials and methods

The primary outcome of the study was in-hospital complications, and the secondary outcome was in-hospital mortality. The study is a retrospective register-based cohort study utilizing the data from the 2013–2019 American College of Surgeons’ Trauma Quality Improvement Program (ACS TQIP) database containing anonymous patient data based on approximately 900 participating level one trauma centers across the United States. All relevant/applicable ethical permits were in place prior to the commencement of this study. The study adhered to the Declaration of Helsinki and the Strengthening the Reporting of Observational Studies in Epidemiology (STROBE) guidelines throughout (11). Information retrieved for this study included age, sex, comorbidities, abbreviated injury scale (AIS), injury pattern, interventions, discharge disposition, and complications. All geriatric patients (65 years or older) who suffered a moderate TBI as a result of a GLF were initially screened for inclusion in the current study. In line with previous literature, a moderate TBI was defined as a head AIS ≤ 3, GCS 9–13 with an AIS ≤ 2 in all other body regions (12, 13). Patients were excluded listwise if they were missing data, in order to facilitate a complete case analysis. These previously listed inclusion and exclusion criteria were selected to better delineate the predictive factors for outcome after TBI, i.e., by excluding severe or significant injuries (AIS 3 and above in all other body regions) unrelated to the TBI.

### 2.1 Statistical analysis

In-hospital complications were defined as myocardial infarction, cardiac arrest with CPR, stroke, deep vein thrombosis, pulmonary embolism, acute respiratory distress syndrome, urinary tract infection, pneumonia, surgical site infection, sepsis, decubitus ulcer, unplanned intubation, unplanned admission to the operating room, and unplanned admission to the intensive care unit. Patients were divided based on if they did or did not experience an in-hospital complication. Continuous data that did not follow a normal distribution were summarized using medians and interquartile ranges, while continuous data that followed a normal distribution was presented as means and standard deviations. The statistical significance of differences in continuous variables was determined using the Mann–Whitney *U*-test for non-normally distributed data and Student’s *t*-test for normally distributed data. Categorical data was summarized with counts and percentages. Differences between these variables were evaluated using the Chi-squared test or Fisher’s exact test, as appropriate.

A logistic regression (LR) model was fitted, with in-hospital complications as the outcome variable and age, sex, injury severity score (ISS), highest AIS in each region, intracranial injuries, neurosurgical intervention, Revised Cardiac Risk Index (RCRI) (14, 15), shock index, vitals on admission to the emergency room (systolic blood pressure, pulse rate, temperature, oxygen saturation, respiratory rate), Glasgow Coma Scale (GCS) on admission to the emergency room, as well as comorbidities (hypertension, history of peripheral vascular disease, functionally dependent health status, chronic obstructive pulmonary disease, smoking status, cirrhosis, coagulopathy, currently receiving chemotherapy for cancer, metastatic cancer, drug use disorder, alcohol use disorder, and major psychiatric illness) as the explanatory variables (16–18). Preadmission anticoagulant therapy was unable to be included as a covariate given over 38% of cases were missing these data. The relative importance of the explanatory variables in predicting in-hospital complications was evaluated using permutation importance (PI) as described by Altmann et al. (19) The PI was calculated by evaluating to what degree a specific value [1 − Area under the receiver-operating characteristic curve (AUC)] was changed by the suppression of a particular variable. Instead of removing each variable from the dataset, the PI method masks each variable’s information by rearranging the variable’s values. This process was repeated 10 times to account for the randomness of permutations. The relative importance of each variable in the model was presented as the average increase in 1-AUC compared to the AUC in a model that included all variables without any permutations. The above steps were also repeated for in-hospital mortality as the outcome in the LR models. While the LR models are used to derive the predictive importance, the coefficients themselves are not presented as they would be biased and lack clinical relevance given the presence of multicollinearity in the models.

Statistical significance was defined as a two-sided *p* < 0.05. The statistical analysis was conducted using statistical programming language R 4.0.5 (R Foundation for Statistical Computing, Vienna, Austria) with the aid of the tidyverse, DALEX, pROC, haven, and cowplot packages (20).

## 3 Results

**Figure 1:**
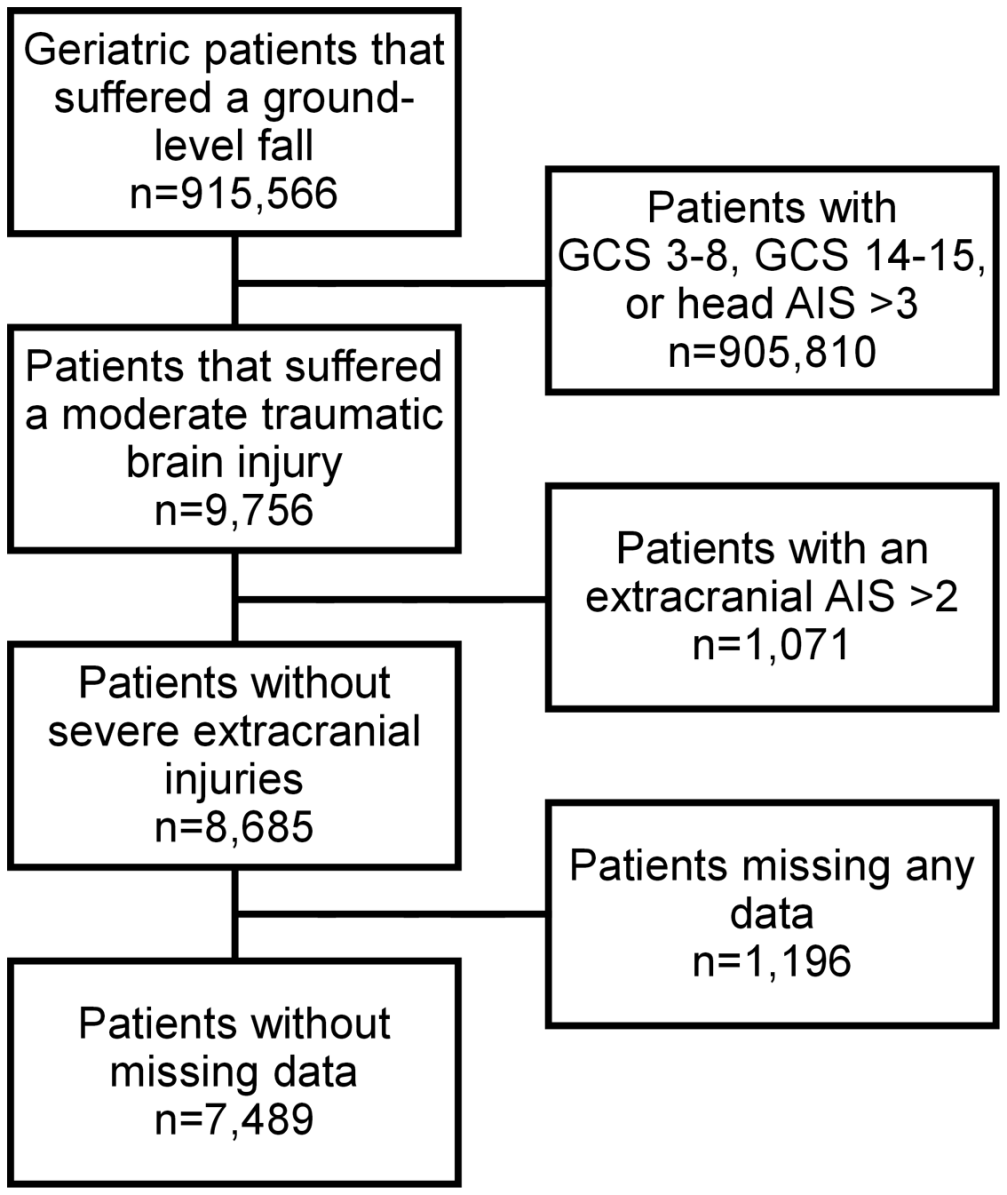
Flow chart describing selection of sample population.

A total of 7,489 patients met the study inclusion criteria (Figure 1). Patients who experienced in-hospital complications were more often male (53.2% vs. 46.9%, *p* = 0.008) and had a higher cardiac risk as measured by the RCRI (RCRI ≥2: 17.3% vs. 9.4%, *p* < 0.001). Most comorbidities were more prevalent among patients who experienced an in-hospital complication (Table 1). This subgroup was more likely to have suffered a more severe head injury (Head AIS 3: 61.8% vs. 44.3%, *p* < 0.001) and present with a lower initial GCS (GCS ≤11: 40.5% vs. 34.0%, *p* = 0.017). This is reflected in the fact that traumatic subdural hematomas (25.9% vs. 16.2%, *p* < 0.001), subarachnoid hemorrhages (24.4% vs. 17.4%, p < 0.001), and cerebral contusions were more common in patients who suffered a complication (15.8% vs. 8.6%, *p* < 0.001), which corresponded to a greater need for neurosurgical intervention (9.4% vs. 1.5%, *p* < 0.001). Patients who experienced a complication were also likelier to be more severely injured overall, being found to frequently have suffered a more severe spine (Spine AIS 2: 11.5% vs. 6.0%, *p* < 0.001) and abdomen injuries (Abdomen AIS 2: 0.6% vs. 0.4%, *p* = 0.024) (Table 2). Similar patterns were observed when comparing the patients who died to those who survived their hospital stay (Supplementary Tables S1, S2). Overall, 6.5% of patients suffered a complication and 6.2% died during their hospital stay (Table 3).

**Table 1:** Demographics of geriatric patients who suffered a moderate TBI as a result of a GLF.

	No complication (*N* = 7,002)	Any complication (*N* = 487)	*p*-value
Age, median [IQR]	79 [73–84]	78 [72–83]	0.030
Sex, n (%)			0.008
Female	3,719 (53.1)	228 (46.8)	
Male	3,283 (46.9)	259 (53.2)	
RCRI*, n (%)			<0.001
0	4,158 (59.4)	233 (47.8)	
1	2,185 (31.2)	170 (34.9)	
2	546 (7.8)	67 (13.8)	
3	104 (1.5)	14 (2.9)	
≥4	9 (0.1)	3 (0.6)	
Hypertension, n (%)	4,269 (61.0)	344 (70.6)	<0.001
Previous myocardial infarction, n (%)	124 (1.8)	11 (2.3)	0.381
Congestive heart failure, n (%)	581 (8.3)	64 (13.1)	<0.001
History of peripheral vascular disease, n (%)	87 (1.2)	11 (2.3)	0.063
Cerebrovascular disease, n (%)	888 (12.7)	71 (14.6)	0.254
Non-independent functional status, n (%)	1,702 (24.3)	92 (18.9)	0.008
Currently receiving chemotherapy for cancer, n (%)	58 (0.8)	7 (1.4)	0.197
Metastatic cancer, n (%)	125 (1.8)	10 (2.1)	0.598
COPD, n (%)	696 (9.9)	70 (14.4)	0.002
Current smoker, n (%)	439 (6.3)	44 (9.0)	0.021
Chronic renal failure, n (%)	245 (3.5)	31 (6.4)	0.002
Diabetes mellitus, n (%)	1,755 (25.1)	157 (32.2)	<0.001
Cirrhosis, n (%)	86 (1.2)	11 (2.3)	0.060
Coagulopathy, n (%)	568 (8.1)	79 (16.2)	<0.001
Drug use disorder, n (%)	114 (1.6)	10 (2.1)	0.598
Alcohol use disorder, n (%)	390 (5.6)	45 (9.2)	0.001
Major psychiatric illness, n (%)	877 (12.5)	68 (14.0)	0.393

*Patients received one point for each of the following: a history of ischemic heart disease, congestive heart failure, cerebrovascular disease, diabetes mellitus, and renal insufficiency (acute kidney injury or chronic kidney disease) (14, 15).TBI, Traumatic brain injury; GLF, Ground-level fall; RCRI, Revised Cardiac Risk Index; COPD, chronic obstructive pulmonary disease.

**Table 2:** Clinical characteristics of geriatric patients who suffered a moderate TBI as a result of a GLF.

	No complication (*N* = 7,002)	Any complication (*N* = 487)	*p*-value
Injury severity score, median [IQR]	8.0 [4.0–10]	9.0 [5.0–10]	<0.001
Head AIS, n (%)			<0.001
1	1,808 (25.8)	91 (18.7)	
2	2,092 (29.9)	95 (19.5)	
3	3,102 (44.3)	301 (61.8)	
Face AIS, n (%)			0.151
Injury not present	4,337 (61.9)	304 (62.4)	
1	2,020 (28.8)	127 (26.1)	
2	645 (9.2)	56 (11.5)	
Neck AIS, n (%)			0.069
Injury not present	6,963 (99.4)	482 (99.0)	
1	33 (0.5)	3 (0.6)	
2	6 (0.1)	2 (0.4)	
Spine AIS, n (%)			<0.001
Injury not present	6,518 (93.1)	429 (88.1)	
1	66 (0.9)	2 (0.4)	
2	418 (6.0)	56 (11.5)	
Thorax AIS, n (%)			0.080
Injury not present	6,655 (95.0)	453 (93.0)	
1	228 (3.3)	20 (4.1)	
2	119 (1.7)	14 (2.9)	
Abdomen AIS, n (%)			0.024
Injury not present	6,875 (98.2)	470 (96.5)	
1	102 (1.5)	14 (2.9)	
2	25 (0.4)	3 (0.6)	
Upper extremity AIS, n (%)			0.638
Injury not present	5,671 (81.0)	390 (80.1)	
1	982 (14.0)	68 (14.0)	
2	349 (5.0)	29 (6.0)	
Lower extremity AIS, n (%)			0.861
Injury not present	6,140 (87.7)	423 (86.9)	
1	691 (9.9)	51 (10.5)	
2	171 (2.4)	13 (2.7)	
External/Other AIS, n (%)			0.460
Injury not present	6,784 (96.9)	469 (96.3)	
1	217 (3.1)	18 (3.7)	
2	1 (0.0)	0 (0.0)	
Epidural hematoma, n (%)	28 (0.4)	5 (1.0)	0.060
Traumatic subdural hematoma, n (%)	1,131 (16.2)	126 (25.9)	<0.001
Traumatic subarachnoid hemorrhage, n (%)	1,219 (17.4)	119 (24.4)	<0.001
Cerebral contusion, n (%)	601 (8.6)	77 (15.8)	<0.001
Diffuse axonal injury, n (%)	18 (0.3)	2 (0.4)	0.377
Neurosurgical intervention, n (%)	106 (1.5)	46 (9.4)	<0.001
Shock index, median [IQR]	0.57 [0.47–0.69]	0.58 [0.48–0.70]	0.228
Systolic blood pressure on admission, mean (SD)	148 (±29.8)	151 (±32.1)	0.039
Pulse rate on admission, mean (SD)	85.3 (±19.4)	88.8 (±20.1)	<0.001
Temperature on admission, median [IQR]	37 [36–37]	37 [36–37]	0.648
Oxygen saturation on admission, median [IQR]	97 [95–99]	97 [95–99]	0.788
Respiratory rate on admission, mean (SD)	18.8 (±4.6)	19.2 (±5.1)	0.043
Glasgow Coma Scale on admission, n (%)			0.017
13	3,259 (46.5)	205 (42.1)	
12	1,358 (19.4)	85 (17.5)	
11	1,080 (15.4)	78 (16.0)	
10	814 (11.6)	69 (14.2)	
9	491 (7.0)	50 (10.3)	

TBI, traumatic brain injury; GLF, ground-level fall; AIS, abbreviated injury severity score.

**Table 3:** Crude outcomes in geriatric patients who suffered a moderate TBI as a result of a GLF.

	No complication (*N* = 7,002)	Any complication (*N* = 487)	*p*-value
In-hospital mortality, n (%)	348 (5.0)	120 (24.6)	<0.001
Any complication, n (%)	0 (0.0)	487 (100.0)	<0.001
Myocardial infarction	0 (0.0)	30 (6.2)	<0.001
Cardiac arrest with CPR	0 (0.0)	47 (9.7)	<0.001
Stroke	0 (0.0)	35 (7.2)	<0.001
DVT	0 (0.0)	42 (8.6)	<0.001
Pulmonary embolism	0 (0.0)	11 (2.3)	<0.001
ARDS	0 (0.0)	19 (3.9)	<0.001
Urinary tract infection	0 (0.0)	75 (15.4)	<0.001
Pneumonia	0 (0.0)	54 (11.1)	<0.001
Surgical site infection	0 (0.0)	4 (0.8)	<0.001
Sepsis	0 (0.0)	32 (6.6)	<0.001
Decubitus ulcer	0 (0.0)	34 (7.0)	<0.001
Unplanned intubation	0 (0.0)	126 (25.9)	<0.001
Unplanned admission to the OR	0 (0.0)	7 (1.4)	<0.001
Unplanned admission to the ICU	0 (0.0)	145 (29.8)	<0.001

TBI, traumatic brain injury; GLF, ground-level fall; DVT, deep vein thrombosis; ARDS, acute respiratory distress syndrome; OR, operating room; ICU, intensive care unit.

**Figure 2:**
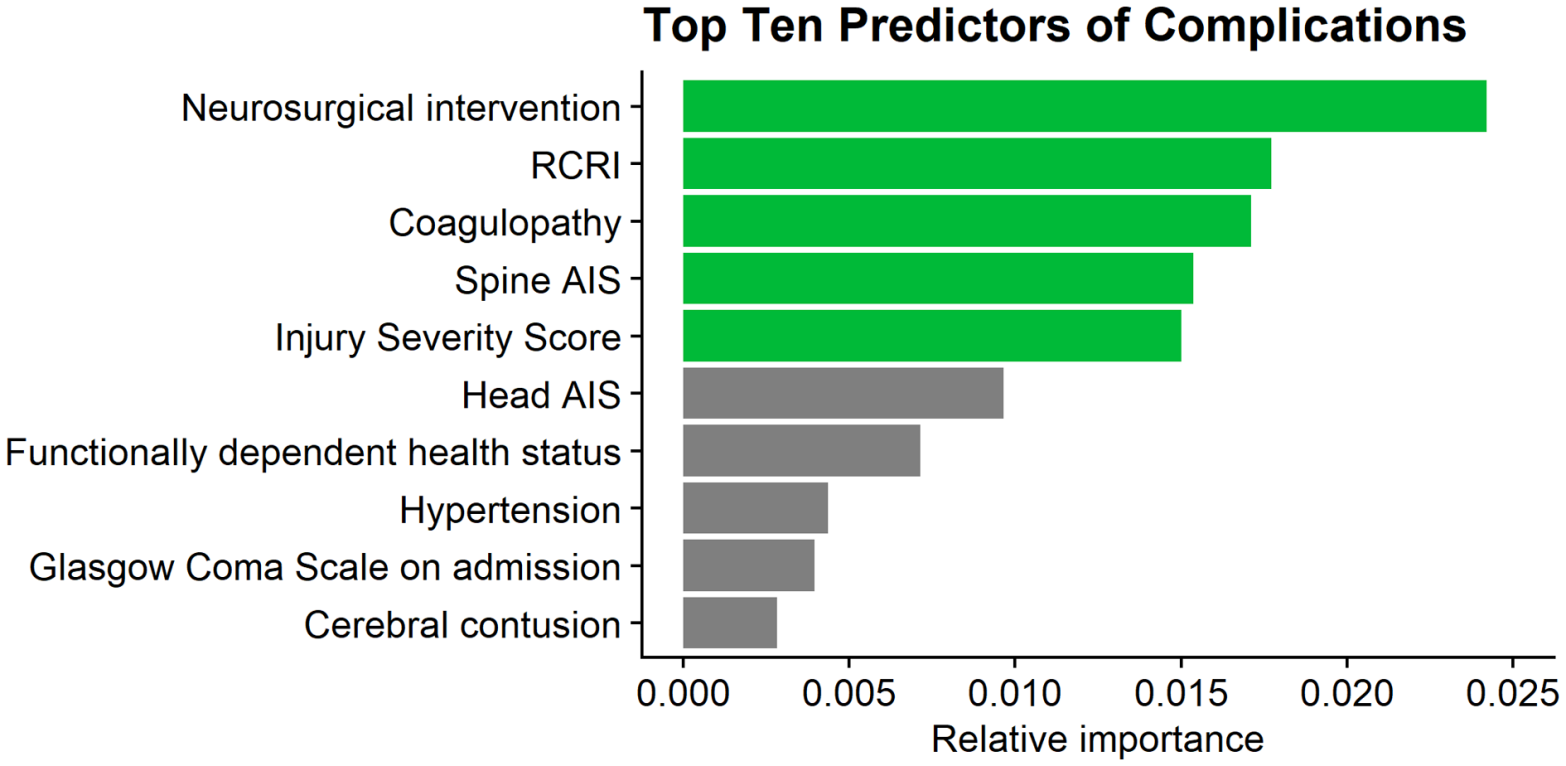
Top 10 predictors of complications. RCRI, Revised Cardiac Risk Index; AIS, abbreviated injury severity score.

The full LR model for in-hospital complications contained a total of 38 variables and resulted in an acceptable predictive ability, with an AUC of 0.71 (95% Confidence Interval: 0.68–0.74) (21). The top five predictors of in-hospital complications in this model were the need for neurosurgical intervention, the RCRI, coagulopathy, spine AIS, and ISS (Figure 2).

**Figure 3:**
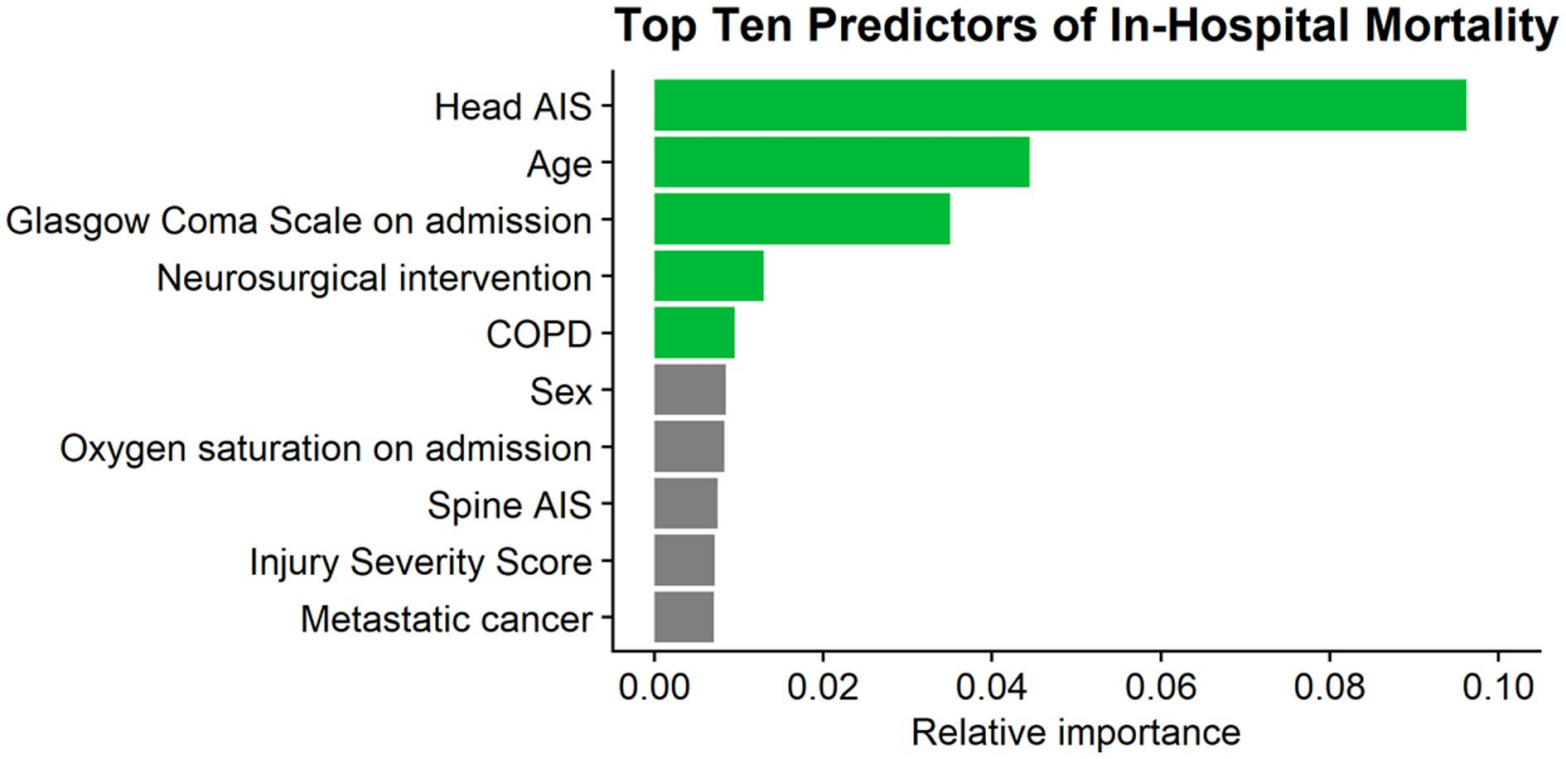
Top 10 predictors of mortality. AIS, abbreviated injury severity score; COPD, chronic obstructive pulmonary disease.

The full LR model for in-hospital mortality contained the same 38 variables and also resulted in an acceptable predictive ability, with an AUC of 0.77 (95% Confidence Interval: 0.75–0.79) (21). The top five predictors of in-hospital mortality in this model were head AIS, age, GCS on admission, the need for neurosurgical intervention, and chronic obstructive pulmonary disease (Figure 3).

## 4 Discussion

This study represents the first of its kind, investigating predictors of complications and mortality in moderate TBIs. Although previous studies, like the one conducted by Dams-O’Connor et al., highlight that the proportion of elderly patients who suffer a moderate TBI following a GLF is smaller (5%) compared to mild and severe TBIs (84%), the actual number of affected elderly individuals would still be quite substantial, amounting to several thousand elderly patients annually in the United States alone (22, 23). Furthermore, while 80–88% of mild TBI patients recover most or all function, approximately 15% of geriatric moderate TBI lose their lives and up to 80% continue to face significant disabilities even after recovery (6, 9, 22).

Despite this significant impact on patients’ lives, the research gap between moderate TBIs and their mild or severe counterparts is vast, leading to a paucity in clear guidelines for effectively treating and managing patients with moderate TBIs (9, 10). In light of this, the current investigation aimed to address this issue by identifying predictors of complications and mortality in geriatric patients with moderate TBIs. Such insights can be invaluable to healthcare professionals, providing them with better tools to assess and manage patients. The need for neurosurgical intervention was found to be the most important predictor of complications, followed by the patients’ RCRI, coagulopathy, spine AIS, and ISS. The best predictors of in-hospital mortality were head AIS, followed by age, GCS on admission, the need for neurosurgical intervention, and chronic obstructive pulmonary disease. The need for neurosurgical intervention uniquely stood out as the only predictor of both complications and mortality.

Contrary to previous research that has shown a link between age and an increased frequency of complications following trauma as well as worse outcomes in moderate TBI, age was not identified as one of the top predictors of complications in this study (8, 24, 25). This difference may be attributable to the exclusive focus on geriatric patients, and beyond a certain threshold, age may have a lesser impact on in-hospital complications. Prior investigations that explored age and complications typically examined wider age ranges. Furthermore, this study did not incorporate frailty as a factor, which could be more important than chronological age alone in predicting patient outcomes. Conversely, hypertension and GCS being among the top 10 most important predictors is in line with previous research, which has found that comorbidities and GCS following injury have a strong association with higher rates of complications (24–26).

While predictors of outcome according to the Glasgow Outcome Scale, and other classifications are more common, there are few studies looking at predictors of in-hospital complications following TBI, and none that specifically examine moderate TBI in isolation. Due to the lack of research focused solely on moderate TBI, the results of this study are compared with others that performed the statistical analysis on different groupings of TBI severity. The association between the need for neurosurgical intervention and complications following a TBI has garnered substantial support in academic literature (27–30). Omar et al., for example, recognized neurosurgical intervention as a top predictor of complications, drawing the same conclusion as the present study (30). This finding was also indirectly echoed by Scheetz, who determined that the most significant predictor of in-hospital complications was a major operating room procedure (31). Additionally, in accordance previous investigations including Omar et al., injury severity was found to hold substantial weight in predicting complications in the current analysis (24, 25, 30). However, these studies included all TBI severities.

The results of this study could have several clinical applications. Primarily, they can be utilized to identify elderly patients who are at higher risk of in-hospital complications or mortality following a TBI. By identifying these high-risk individuals, healthcare providers can allocate resources more efficiently and design tailored care plans to address their specific needs. Targeted interventions can be implemented to minimize the risks altogether, leading to improved TBI outcomes. One such intervention, supported by previous research, involves the use of beta-blockers. Studies have demonstrated that beta-blockers can effectively reduce cardiac complications, which are a primary extracranial cause of death associated with TBI, and even decrease mortality following severe TBI (32, 33). Notably, approximately 20% of patients exhibit cardiac pathology as revealed by post-mortem examinations; this damage closely resembles that found in individuals suffering from conditions like pheochromocytoma or cocaine overdose (34). In addition to cardiac considerations, closely monitoring intracranial physiological variables, such as intracranial pressure and cerebral perfusion pressure, could be critical. This is especially important given the close correlation between neurological intervention and complications (35).

Furthermore, the results of this study could serve as a valuable foundation for developing clinical decision-making tools, such as risk prediction models, to aid in the care of geriatric patients with moderate TBI. By leveraging the insights gained from this research, healthcare professionals can make more informed decisions and tailor treatment plans to individual patients’ needs. This, in turn, may lead to the creation of best practice guidelines specifically designed for this vulnerable patient population. One such tool for identifying at-risk patients is the RCRI, which was found to be the second most important predictor of complications. The RCRI relies on six objective data points that can be readily obtained during admission without requiring invasive tests. Early identification of high-risk patients using the RCRI can facilitate appropriate allocation of resources and expertise, ensuring that patients receive the most suitable and timely interventions (14, 15, 36).

This study possesses several notable strengths that contribute to its credibility and potential impact. One of the most significant strengths is the substantial sample size obtained from a national database, namely the ACS TQIP dataset. This large sample size helps mitigate the risk of random errors and enhances the generalizability of the findings to a broader population of trauma patients in the United States. Additionally, the utilization of the ACS TQIP dataset, a comprehensive database specifically designed to capture trauma patient data, minimizes the potential for selection bias, thus increasing the study’s external validity. However, there are limitations that warrant careful consideration. While the study identifies certain variables as potential predictors of future complications or mortality, it is crucial to note that these associations do not imply causality between the variables and the outcomes. Instead, the findings highlight potential correlations that warrant further investigation into the complex interplay of these factors in influencing patient outcomes following a TBI. The study’s scope was also limited to the variables available in the database, which meant that detailed information regarding computerized tomography findings, indications for surgical intervention, and other possibly important variables were unavailable. Moreover, as with any retrospective study design, there is a risk of non-differential misclassification arising from errors in data entry. Data may also be biased toward specific demographics or conditions, leading to a lack of heterogeneity and potentially limiting the generalizability of the results. Finally, as the data stems from multiple sources, differences in data collection and recording practices across sources can introduce inconsistencies. Preadmission anticoagulant therapy, a potentially critical covariate influencing patient outcomes, was also excluded, as approximately 38% of cases lacked this data. Furthermore, the absence of frailty as a factor in the dataset is another potential limitation. The inclusion of frailty as an assessment tool, such as the Clinical Frailty Scale, could have provided valuable insights into patient outcomes. Research has consistently demonstrated that frailty is a more accurate predictor of outcomes than age alone across various fields of study, underscoring the significance of its inclusion in contexts like this study (37, 38).

## 5 Conclusion

The findings from our study reveal important predictors for in-hospital complications and mortality in geriatric patients with moderate traumatic brain injuries following ground level falls. However, to maximize the impact of these findings, further investigations are warranted. Future studies should explore how these predictors can be integrated into clinical decision-making tools, such as risk prediction models, to assist healthcare professionals in assessing and managing geriatric patients with moderate traumatic brain injury effectively. Additionally, the development of evidence-based guidelines tailored specifically for moderate TBI treatment in this population is imperative.

## Data availability statement

The datasets presented in this article are not readily available because due to TQIP data restrictions by American College of Surgeons. Requests to access the datasets should be directed to mohsenishahin@yahoo.com and/or the American College of Surgeons.

## Ethics statement

Ethical approval was not required for the study involving humans in accordance with the local legislation and institutional requirements. Written informed consent to participate in this study was not required from the participants or the participants’ legal guardians/next of kin in accordance with the national legislation and the institutional requirements.

## Author contributions

SF: Formal analysis, Investigation, Writing – original draft, Methodology. RA: Writing – original draft, Formal analysis, Conceptualization, Investigation, Supervision. MF: Writing – review & editing, Formal analysis, Methodology, Data curation, Software, Visualization. MR: Writing – review &a editing, Formal analysis, Conceptualization, Investigation, Supervision, Validation. BS: Writing – review & editing, Data curation, Formal analysis, Project administration, Resources, Supervision, Validation. SM: Project administration, Supervision, Writing – original draft, Data curation, Methodology, Conceptualization, Funding acquisition, Investigation, Resources, Validation.
