# Supplementary material for: Predictors of outcomes in geriatric patients with moderate traumatic brain injury after ground level falls
Source: Front Med (Lausanne). 2023 Dec 12;10:1290201. doi: 10.3389/fmed.2023.1290201 (PMC10751787; doi:10.3389/fmed.2023.1290201)
Supplement: Supplementary file 1 [file Table_1.DOCX]

| **Supplemental Table 1.** Demographics of geriatric patients who suffered a moderate TBI as a result of a GLF | | | |
| --- | --- | --- | --- |
|  | **Survived (N = 7,021)** | **Died (N = 468)** | **P-value** |
| Age, median [IQR] | 79 [72-84] | 82 [75-86] | <0.001 |
| Sex, n (%) |  |  | <0.001 |
| Female | 3,744 (53.3) | 203 (43.4) |  |
| Male | 3,277 (46.7) | 265 (56.6) |  |
| RCRI, n (%) |  |  | <0.001 |
| 0 | 4,145 (59.0) | 246 (52.6) |  |
| 1 | 2,210 (31.5) | 145 (31.0) |  |
| 2 | 554 (7.9) | 59 (12.6) |  |
| 3 | 103 (1.5) | 15 (3.2) |  |
| ≥4 | 9 (0.1) | 3 (0.6) |  |
| Hypertension, n (%) | 4,306 (61.3) | 307 (65.6) | 0.074 |
| Previous myocardial infarction, n (%) | 126 (1.8) | 9 (1.9) | 0.857 |
| Congestive heart failure, n (%) | 571 (8.1) | 74 (15.8) | <0.001 |
| History of peripheral vascular disease, n (%) | 86 (1.2) | 12 (2.6) | 0.020 |
| Cerebrovascular disease, n (%) | 889 (12.7) | 70 (15.0) | 0.172 |
| Non-independent functional status, n (%) | 1,693 (24.1) | 101 (21.6) | 0.235 |
| Currently receiving chemotherapy for cancer, n (%) | 58 (0.8) | 7 (1.5) | 0.124 |
| Metastatic cancer, n (%) | 112 (1.6) | 23 (4.9) | <0.001 |
| COPD, n (%) | 685 (9.8) | 81 (17.3) | <0.001 |
| Current smoker, n (%) | 447 (6.4) | 36 (7.7) | 0.301 |
| Chronic renal failure, n (%) | 249 (3.5) | 27 (5.8) | 0.019 |
| Diabetes mellitus, n (%) | 1,787 (25.5) | 125 (26.7) | 0.583 |
| Cirrhosis, n (%) | 85 (1.2) | 12 (2.6) | 0.019 |
| Coagulopathy, n (%) | 582 (8.3) | 65 (13.9) | <0.001 |
| Drug use disorder, n (%) | 118 (1.7) | 6 (1.3) | 0.640 |
| Alcohol use disorder, n (%) | 412 (5.9) | 23 (4.9) | 0.452 |
| Major psychiatric illness, n (%) | 893 (12.7) | 52 (11.1) | 0.346 |
| *TBI, Traumatic brain injury; GLF, Ground-level fall; RCRI, Revised Cardiac Risk Index; COPD, chronic obstructive pulmonary disease* | | | |

| **Supplemental Table 2.** Clinical characteristics of geriatric patients who suffered a moderate TBI as a result of a GLF | | | |
| --- | --- | --- | --- |
|  | **Survived (N = 7,021)** | **Died (N = 468)** | **P-value** |
| Injury Severity Score, median [IQR] | 8.0 [4.0-10] | 9.0 [6.0-10] | <0.001 |
| Head AIS, n (%) |  |  | <0.001 |
| 1 | 1,832 (26.1) | 67 (14.3) |  |
| 2 | 2,102 (29.9) | 85 (18.2) |  |
| 3 | 3,087 (44.0) | 316 (67.5) |  |
| Face AIS, n (%) |  |  | 0.029 |
| Injury not present | 4,330 (61.7) | 311 (66.5) |  |
| 1 | 2,038 (29.0) | 109 (23.3) |  |
| 2 | 653 (9.3) | 48 (10.3) |  |
| Neck AIS, n (%) |  |  | 0.093 |
| Injury not present | 6,980 (99.4) | 465 (99.4) |  |
| 1 | 35 (0.5) | 1 (0.2) |  |
| 2 | 6 (0.1) | 2 (0.4) |  |
| Spine AIS, n (%) |  |  | 0.043 |
| Injury not present | 6,526 (92.9) | 421 (90.0) |  |
| 1 | 63 (0.9) | 5 (1.1) |  |
| 2 | 432 (6.2) | 42 (9.0) |  |
| Thorax AIS, n (%) |  |  | 0.012 |
| Injury not present | 6,677 (95.1) | 431 (92.1) |  |
| 1 | 223 (3.2) | 25 (5.3) |  |
| 2 | 121 (1.7) | 12 (2.6) |  |
| Abdomen AIS, n (%) |  |  | 0.291 |
| Injury not present | 6,889 (98.1) | 456 (97.4) |  |
| 1 | 105 (1.5) | 11 (2.4) |  |
| 2 | 27 (0.4) | 1 (0.2) |  |
| Upper extremity AIS, n (%) |  |  | 0.094 |
| Injury not present | 5,699 (81.2) | 362 (77.4) |  |
| 1 | 969 (13.8) | 81 (17.3) |  |
| 2 | 353 (5.0) | 25 (5.3) |  |
| Lower extremity AIS, n (%) |  |  | 0.117 |
| Injury not present | 6,162 (87.8) | 401 (85.7) |  |
| 1 | 693 (9.9) | 49 (10.5) |  |
| 2 | 166 (2.4) | 18 (3.8) |  |
| External/Other AIS, n (%) |  |  | 0.215 |
| Injury not present | 6,805 (96.9) | 448 (95.7) |  |
| 1 | 215 (3.1) | 20 (4.3) |  |
| 2 | 1 (0.0) | 0 (0.0) |  |
| Epidural hematoma, n (%) | 30 (0.4) | 3 (0.6) | 0.459 |
| Traumatic subdural hematoma, n (%) | 1,141 (16.3) | 116 (24.8) | <0.001 |
| Traumatic subarachnoid hemorrhage, n (%) | 1,214 (17.3) | 124 (26.5) | <0.001 |
| Cerebral contusion, n (%) | 596 (8.5) | 82 (17.5) | <0.001 |
| Diffuse axonal injury, n (%) | 19 (0.3) | 1 (0.2) | 1.00 |
| Neurosurgical intervention, n (%) | 117 (1.7) | 35 (7.5) | <0.001 |
| Shock index, median [IQR] | 0.57 [0.47-0.69] | 0.58 [0.47-0.71] | 0.323 |
| Systolic blood pressure on admission, mean (SD) | 148 (±29.8) | 149 (±32.4) | 0.578 |
| Pulse rate on admission, mean (SD) | 85.4 (±19.3) | 87.5 (±21.8) | 0.042 |
| Temperature on admission, median [IQR] | 37 [36-37] | 37 [36-37] | 0.057 |
| Oxygen saturation on admission, median [IQR] | 97 [95-99] | 96 [94-99] | <0.001 |
| Respiratory rate on admission, mean (SD) | 18.7 (±4.6) | 19.7 (±4.7) | <0.001 |
| Glasgow Coma Scale on admission, n (%) |  |  | <0.001 |
| 13 | 3,309 (47.1) | 155 (33.1) |  |
| 12 | 1,362 (19.4) | 81 (17.3) |  |
| 11 | 1,080 (15.4) | 78 (16.7) |  |
| 10 | 805 (11.5) | 78 (16.7) |  |
| 9 | 465 (6.6) | 76 (16.2) |  |
| *TBI, Traumatic brain injury; GLF, Ground-level fall; AIS, Abbreviated injury severity score* | | | |
